# Supplementary material for: Optimal minimal residual disease threshold in pediatric acute myeloid leukemia: A retrospective cohort study based on the TARGET database
Source: PLoS Med. 2026 May 8;23(5):e1005088. doi: 10.1371/journal.pmed.1005088 (PMC13155632; doi:10.1371/journal.pmed.1005088)
Supplement: S1 Code — (ZIP) [file pmed.1005088.s002.zip › S2 code/PROJ8_9_tbl/PROJ8_9_tbl.htm]

## ¶à¸ö»Ø¹é·½³Ì

|  |  |  |
| --- | --- | --- |
| Exposure | Univariable | Multivariable |
| Gender |  |  |
| Male | 1.0 | 1.0 |
| Female | 0.8 (0.7, 1.0) 0.021 | 0.8 (0.6, 1.1) 0.169 |
| Age group |  |  |
| £¼10 | 1.0 | 1.0 |
| ¡Ý10 | 1.0 (0.8, 1.1) 0.809 | 1.4 (1.0, 1.9) 0.041 |
| Chemotherapy Protocol |  |  |
| AAML1031 | 1.0 | 1.0 |
| AAML0531 | 1.0 (0.9, 1.2) 0.602 | 1.0 |
| AAML03P1 | 0.8 (0.5, 1.2) 0.217 | 0.8 (0.5, 1.2) 0.244 |
| Risk group |  |  |
| Low risk | 1.0 | 1.0 |
| Standard risk | 2.8 (2.3, 3.3) <0.001 | 3.1 (1.2, 8.3) 0.022 |
| High risk | 2.7 (2.0, 3.5) <0.001 | 1.9 (0.7, 5.3) 0.198 |
| WBC | 1.0 (1.0, 1.0) <0.001 | 1.0 (1.0, 1.0) 0.154 |
| BM blast(%) | 1.0 (1.0, 1.0) 0.003 | 1.0 (1.0, 1.0) 0.298 |
| Peripheral blasts (%) | 1.0 (1.0, 1.0) 0.006 | 1.0 (1.0, 1.0) 0.080 |
| Karyotype |  |  |
| MLL | 1.0 | 1.0 |
| t(8;21) | 0.4 (0.3, 0.5) <0.001 | 0.9 (0.3, 2.9) 0.862 |
| inv(16) | 0.5 (0.4, 0.7) <0.001 | 1.3 (0.4, 4.1) 0.614 |
| Normal | 0.8 (0.6, 1.0) 0.024 | 1.5 (0.8, 2.8) 0.255 |
| Other | 1.1 (0.9, 1.3) 0.451 | 1.3 (0.8, 2.2) 0.329 |
| FLT3-ITD Status |  |  |
| FLT3-ITD-wt | 1.0 | 1.0 |
| FLT3-ITD-mt | 1.4 (1.1, 1.7) 0.002 | 1.0 (0.6, 1.7) 0.935 |
| NPM1 Status |  |  |
| NPM1-wt | 1.0 | 1.0 |
| NPM1-mt | 0.4 (0.3, 0.6) <0.001 | 1.6 (0.6, 4.2) 0.342 |
| CEBPA Status |  |  |
| CEBPA-wt | 1.0 | 1.0 |
| CEBPA-mt | 0.6 (0.4, 0.9) 0.011 | 0.8 (0.2, 2.7) 0.674 |
| WT1 Status |  |  |
| WT1-wt | 1.0 | 1.0 |
| WT1-mt | 2.1 (1.4, 3.0) <0.001 | 1.8 (1.1, 3.2) 0.029 |
| FAB Category |  |  |
| M0 | 1.0 | 1.0 |
| M1 | 0.4 (0.3, 0.7) <0.001 | 0.3 (0.1, 0.8) 0.011 |
| M2 | 0.3 (0.2, 0.4) <0.001 | 0.5 (0.2, 1.1) 0.070 |
| M4 | 0.4 (0.3, 0.6) <0.001 | 0.6 (0.3, 1.3) 0.188 |
| M5 | 0.5 (0.4, 0.8) 0.002 | 0.4 (0.2, 0.8) 0.012 |
| M6 | 0.6 (0.2, 1.3) 0.181 | 0.1 (0.0, 1.0) 0.052 |
| M7 | 0.4 (0.2, 0.7) <0.001 | 0.3 (0.1, 0.8) 0.014 |
| Chloroma |  |  |
| No | 1.0 | 1.0 |
| Yes | 1.2 (0.9, 1.6) 0.164 | 1.0 (0.7, 1.6) 0.891 |
| MRD at end of course 1 |  |  |
| £¼0.1 | 1.0 | 1.0 |
| ¡Ý0.1 | 2.7 (2.3, 3.2) <0.001 | 0.2 (0.1, 0.8) 0.023 |
| MRD at end of course 2 |  |  |
| £¼0.1 | 1.0 | 1.0 |
| ¡Ý0.1 | 3.7 (3.0, 4.6) <0.001 | 1.7 (0.4, 7.3) 0.458 |
| SCT in 1st CR |  |  |
| No | 1.0 | 1.0 |
| Yes | 1.1 (0.9, 1.3) 0.536 | 0.6 (0.4, 0.9) 0.024 |
| MRD % at end of course 1 ·Ö×é |  |  |
| <0.05 | 1.0 | 1.0 |
| >=0.05 | 2.8 (2.3, 3.3) <0.001 | 6.7 (1.8, 24.0) 0.004 |
| MRD % at end of course 2 ·Ö×é |  |  |
| <0.05 | 1.0 | 1.0 |
| >=0.05 | 3.7 (3.0, 4.6) <0.001 | 1.0 (0.2, 3.9) 0.979 |

±íÖÐÊý¾Ý£ºHR (95% CI) Pvalue \*P<0.05 \*\*P<0.01 \*\*\*P<0.001
½á¹û±äÁ¿: First Event
±©Â¶±äÁ¿: Gender; Age group; Chemotherapy Protocol; Risk group; WBC; BM blast(%); Peripheral blasts (%); Karyotype; FLT3-ITD Status; NPM1 Status; CEBPA Status; WT1 Status; FAB Category; Chloroma; MRD at end of course 1; MRD at end of course 2; SCT in 1st CR; MRD % at end of course 1 ·Ö×é; MRD % at end of course 2 ·Ö×é
 Non-adjusted model adjust for: None
Cox model Ê±¼ä±äÁ¿:
X28
´Ë±íÓÃÒ×õÍ³¼ÆÈí¼þ (www.empowerstats.com) ºÍRÈí¼þÉú³É£¬Éú³ÉÈÕÆÚ£º 2025-10-07
¸÷Ä£ÐÍËùÓÃµÄÑù±¾Á¿

|  |  |  |  |  |
| --- | --- | --- | --- | --- |
| Outcome | Model | Exposure | Univariable | Multivariable |
| First Event | Non-adjusted | Gender | 1205 | 396 |
| First Event | Non-adjusted | Age group | 1205 | 396 |
| First Event | Non-adjusted | Chemotherapy Protocol | 1205 | 396 |
| First Event | Non-adjusted | Risk group | 1185 | 396 |
| First Event | Non-adjusted | WBC | 1205 | 396 |
| First Event | Non-adjusted | BM blast(%) | 1138 | 396 |
| First Event | Non-adjusted | Peripheral blasts (%) | 1190 | 396 |
| First Event | Non-adjusted | Karyotype | 1181 | 396 |
| First Event | Non-adjusted | FLT3-ITD Status | 1205 | 396 |
| First Event | Non-adjusted | NPM1 Status | 1201 | 396 |
| First Event | Non-adjusted | CEBPA Status | 1199 | 396 |
| First Event | Non-adjusted | WT1 Status | 532 | 396 |
| First Event | Non-adjusted | FAB Category | 892 | 396 |
| First Event | Non-adjusted | Chloroma | 845 | 396 |
| First Event | Non-adjusted | MRD at end of course 1 | 1205 | 396 |
| First Event | Non-adjusted | MRD at end of course 2 | 1205 | 396 |
| First Event | Non-adjusted | SCT in 1st CR | 1121 | 396 |
| First Event | Non-adjusted | MRD % at end of course 1 ·Ö×é | 1205 | 396 |
| First Event | Non-adjusted | MRD % at end of course 2 ·Ö×é | 1205 | 396 |
